# microRNA therapies in cancer

**DOI:** 10.1186/2052-8426-2-7

**Published:** 2014-03-04

**Authors:** Sacha I Rothschild

**Affiliations:** Department Internal Medicine, Medical Oncology, University Hospital Basel, Basel, Switzerland

**Keywords:** microRNA, miRNA, Cancer, Oncogene, Tumor suppressor, Therapy

## Abstract

MicroRNAs (miRNAs or miRs) are a family of small non-coding RNA species that have been implicated in the control of many fundamental cellular and physiological processes such as cellular differentiation, proliferation, apoptosis and stem cell maintenance. miRNAs regulate gene expression by the sequence-selective targeting of mRNAs, leading to translational repression or mRNA degradation. Some microRNAs have been categorized as “oncomiRs” as opposed to “tumor suppressor miRs” Modulating the miRNA activities may provide exciting opportunities for cancer therapy. This review highlights the latest discovery of miRNAs involved in carcinogenesis as well as the potential applications of miRNA regulations in cancer treatment. Several studies have demonstrated the feasibility of restoring tumor suppressive miRNAs and targeting oncogenic miRNAs for cancer therapy using *in vivo* model systems.

## Review

### Introduction

In 1993, Lee and coworkers described that a small non-coding RNA was able to regulate the expression and function of another protein-coding RNA [1]. This change in paradigms has made a profound impact in our current understanding of gene regulation. miRNAs are usually 18–25 nucleotides long and highly conserved during evolution (reviewed in [2]) miRNA genes are generally transcribed by RNA polymerase II into long primary transcripts, up to several kilobases (pri-miRNA) [3]. These are subsequently processed in the nucleus by a microprocessor complex, which contains the RNase III enzyme Drosha [4] and DGCR8 [5] to become the so-called pre-miR (around 70 nucleotides long). These precursors are exported by exportin 5 and a RanGTP [6, 7] from the nucleus to the cytoplasm where they are bound to the RNase III enzyme Dicer and the RNA-induced silencing complex (RISC) [8]. RISC is composed of the transactivation-responsive RNA-binding protein (TRBP) and Argonaute 2 (Ago2) [9, 10]. Ago2 cleaves the pre-miRNA 12 nucleotides from its 3’ end and then the Dicer cleaves the Ago2-cleaved precursor miRNA into the double-stranded miRNA [11]. While the active mature strand is retained in RISC, the passenger strand is removed and degraded [12] miRNAs mainly bind to the 3’ untranslated region (UTR) of their target mRNAs. However, recent studies have reported that miRNAs also bind to 5’UTR [13], or open reading frame (ORF) [14] of the target mRNA. By binding to their target mRNA, miRNAs regulate the translation of proteins from mRNA or degrade the mRNA itself [9–11]. Depending on the degree of homology to their 3’UTR target sequence, miRNAs induce translational repression or degradation of mRNAs. miRNAs modulate gene expression impacting all cell functions, including apoptosis, proliferation, cell cycle, differentiation, stem cell maintenance and metabolism [15]. It is estimated that more than 1000 miRNAs are transcribed and that 30% of the human genome is under miRNA regulation, one miRNA being able to modulate post-transcriptionally hundreds of downstream genes.

## MicroRNAs and cancer

More than half of the miRNAs genes are located in cancer-associated genomic regions or in fragile sites. Specific miRNA signatures have been associated with distinct subsets of solid tumors and hematological malignancies [15] miRNAs can act as tumor suppressors when their function loss can initiate or contribute to the malignant transformation of a normal cell. The loss of function of a miRNA could be due to several mechanisms, including genomic deletion, mutation, epigenetic silencing, and/or miRNA processing alterations [16–19]. On the other hand, miRNAs can act as oncogenic microRNAs by targeting mRNAs encoding tumor suppressor proteins.

The let-7 family of miRNAs is a typical tumor suppressor and is therefore downregulated in many tumors, including lung and breast cancer [20, 21]. Many of the let-7 family members are located in fragile genomic areas associated with lung, breast, and cervical cancer [22]. Furthermore, let-7 family members functionally inhibit the mRNAs of well-characterized oncogenes, such as RAS [23, 24], HMGA2 [25], and c-Myc [26]. The miR-29 family comprises three isoforms arranged in two clusters: miR-29b-1/miR-29a in chromosome 7q32 and miR-29b-2/miR-29c in chromosome 1q23. miR-29 family members have been shown to b downregulated in chronic lymphatic leukemia (CLL), acute myeloid leukemia (AML), lung cancer, breast cancer, and cholangiocarcinoma [17, 20, 21, 27, 28].

miR-155 was one of the first described oncogenic miRNAs [29, 30] and it is highly expressed in a variety of tumors [17, 20, 21, 28–31]. The miR-155 gene is located in chromosome 21q23 embedded in a host noncoding RNA named the B cell integration cluster (BIC) [32]. BIC is known to cooperate with c-Myc in oncogenesis. Another widely expressed miRNA in hematopoietic and solid tumors is miRNA-21 [17, 28, 31, 33–35] miR-21 targets several tumor suppressor genes such as phosphatase and tensin homolog (PTEN) [34], programmed cell death 4 (PDCD4) [36], and tropomyosin 1 (TPM1) [37]. The miR-17-92 cluster (miR-17, miR-18a, miR-19a, miR-20a, miR-19b-1, miR-92-1) is located at 13q31.3 in a region that is frequently amplified in follicular lymphoma and diffuse large B cell lymphoma [38]. Members of the miR-17-92 cluster are highly expressed in a variety of solid tumors and hematological malignancies [39], Interestingly, the miR-17-92 cluster is transactivated by c-Myc, a frequently activated oncogene in cancer [40].

Recently, miRNAs have been found to foster tumor progression by the mediation of inflammation processes through regulation of components of the innate immune system. Two recent studies described the miRNAs miR-21 and miR-29a to serve as ligands for Toll-like receptor (TLR) activation. Fabbri *et al.* showed that tumor-originating extracellular miRNA could bind to murine TLR7 and human TLR8 to cause a proinflammatory response leading to tumor progression both in vitro and in vivo [41]. In a separate study, Lehmann *et al.* showed that extracellular let-7 could activate TLR7 to induce neurodegeneration [42]. These off-target effects might be overcome by chemical modifications and improved delivery systems as discussed in one of the subsequent paragraphs.

## Targeting microRNAs in cancer

### General aspects of miRNA therapeutics

Every miRNA has multiple target sites in different genes (on average about 500 for each miRNA family). Reciprocally, about two third of all mRNAs have one or more evolutionarily conserved sequences that are predicted to interact with miRNAs [43–46]. The rationale for using miRNAs as therapeutic agents is based on the two following criteria. (1) miRNA expression is dysregulated in cancer compared to normal tissues and (2) the cancer phenotype can be changed by targeting miRNA expression [16, 20, 21, 24, 28, 47–50]. Compared to other strategies, miRNA-based therapeutics have several advantages, as for example the fact that miRNAs as therapeutic agents have the ability to target multiple genes, frequently in the context of a network. The challenges for microRNA-based are the same as the challenges for small interfering RNA therapeutics and include issues of delivery, potential off-target effects and safety. One of the major obstacles for the use of miRNA therapeutics is the tissue-specific delivery [51, 52]. Moreover, the fact that one miRNA targets multiple genes is also a drawback as the potential off-target effects may cause toxic phenotypes [51, 53]. The fact that some biological functions of miRNAs may be partially redundant, or cell-type dependent, is another relevant issue in the development of miRNA therapeutics [54]. Although successful delivery is an obstacle to effective miRNA-based therapeutics, new findings from recent trials and the rapid advances in systemic drug delivery systems provide an optimistic perspective on the progress in this field [55].

In general, miRNA therapeutic approaches can be divided into two different categories: (1) miRNA inhibition therapy when the target miRNA is overexpressed and (2) miRNA replacement therapy when the miRNA is repressed. Therapeutic targeting of microRNAs can be accomplished either by direct inhibition or replacement of miRNAs or by targeting specific genes and therefore regulating the expression of specific miRNAs. For this purpose small-interfering RNAs (siRNAs) and genetically encoded expression vectors encoding small hairpin RNAs (shRNAs) are used [56].

### microRNA modifications

There are different hurdles to develop miRNA-based treatment approaches. One is that RNAs in general have low stability *in vivo*. Thus, miRNA introduced into mice via the tail vein is cleared from the circulatory system within 30 minutes [57]. Unmodified RNAs undergo degradation by RNases and then rapid renal excretion [58]. Therefore, the plasma half-life of RNAs needs to be increased for clinical use of miRNA-based therapeutic approaches. An improvement could be reached by higher miRNA stability or by protection from RNases. By using chemically modified oligonucleotides the stability of the antisense sequences is augmented [59, 60]. In the following established chemical modifications are listed: locked nucleic acid (LNA) oligonucleotides [61], phosphorothioate containing oligonucleotides [62], 2’–O-methyl-(2’–O-Me) or 2’–O-methoxyethyl-oligonucleotides (2’–O-MOE) [63], peptide nucleic acids (PNA) [64], fluorine derivatives (FANA and 2’-F) and other chemical modifications [65]. LNAs are oligonucleotides complementary to the sequence of the targeted mature miRNA, but biochemically modified to reduce the risk of degradation by cellular RNases. LNAs have an extra bridge connecting the 2’ oxygen and the 4’ carbon. PNAs are uncharged oligonucleotides analogs in which the sugar-phosphate backbone is replaced by an achiral structure consisting of N-(2-aminoethyl)-glycine units [66–70]. These molecules form a double helix by hybridization with complementary DNA and RNA [66, 67] PNAs have a higher affinity to RNA than to DNA and are resistant to DNases and proteases [67]. This technology has been investigated in different studies [70, 71] PNA targeting miR-221 has shown to specifically interact with miR-221 expressed in aggressive breast cancer cell lines [72] PNA-anti-miR-21 has recently been shown to reduce breast cancer metastasis in an animal model [73]. It is well established that cancers are often driven by deregulation of various miRNAs or families of miRNAs. Therefore, methods to silence multiple miRNAs have been investigated. Tiny 8-mer LNAs with a phosphorothioate backbone to enhance stability have been developed [74].

### miRNA delivery systems

Beside chemical modification of miRNAs to avoid rapid degradation and excretion, the development of improved delivery systems leads to enhanced stability and more precise delivery of miRNAs. miRNAs can be conjugated to a cholesterol moiety, increasing stability in the circulation and facilitating cell entry [75]. A further mechanism of protection is to enclose miRNA mimics or LNAs into nanoparticles to form micelle-like structures. Liposome-nanoparticles are phospholipid structures that are capable of incorporating various types of nucleic acids and charged small molecules, such as microRNAs, siRNAs, shRNAs, plasmid DNA, and protein, within the aqueous core of the liposome [76]. The major drawback of liposomes are nonspecific uptake and induction of immune response [77]. Polycationic liposome-hyaluronic acid (LPH) nanoparticles have also been used as miRNA carriers [78]. Using LPH particles as a carrier for miR-34a significantly reduced lung metastases in a murine melanoma model [76]. It has been shown that systemic administration of positively charged lipid nanoparticles in vivo is toxic and stimulates inflammatory response by elevating both Th1 and Th17 cytokines and interferon responsive genes [79]. Clearly, a complete understanding of the best liposomal design for delivery of therapeutic substances is still evolving. It is possible that with nucleic acid delivery the use of a neutral lipid, such as 1,2-Dioleoyl-sn-glycero-3-phosphatidylcholine (DOPC) will have several advantages [80]. Other delivery systems used for microRNAs are polyethylenimine (PEI)-based systems [81–83], dendrimers [84–86], poly(lactide-co-glycolide) (PLGA) particles [87, 88], protamine [89], atelocollagen [90–92], as well as inorganic materials (e.g. gold [93, 94] and silica-based nanoparticles [95]).

Another hurdle in the design and application of miRNA therapeutics is to ensure tumor-specific delivery. Due to the fact that most miRNAs target many different mRNAs, off-target effects are a substantial problem. Targeted delivery to specific tissues can be achieved by binding tumor-specific ligands to nanoparticles, which can be directed to tumor cells via active or passive targeting. Active targeting is achieved by conjugation with different compounds that have a specific affinity to tumors. As an example, cancer cell receptors (EGFR, HER-2) or hyaluronic acid could be used [96–98]. Hyaluronic acid is a polysaccharide that binds to the cancer stem cell marker CD44, which is overexpressed in various tumor cells [99].

### miRNA inhibition therapy

Oncogenic microRNAs could be therapeutically targeted by repression and therefore inhibition of the interaction between miRNA and mRNA. A simple method to inhibit miRNAs is the use of oligonucleotides complementary to the mature miRNA (antagomiRs). These oligonucleotides disrupt the miRISC complex and therefore prevent the degradation of the mRNA which can then be translated. microRNA sponges have been developed to inhibit the activity of miRNA families sharing a common seed sequence. miRNA sponges work with multiple complementary 3’UTR mRNA sites of a specific miRNA and saturate the miRISC complex repressing the activity toward natural mRNA [100]. A major drawback of miRNA sponges is the limited homogeneity of transcripts expression and therefore miRNA sponges could lead to serious side effects [101]. Another approach to more specifically inhibit the miRNA function is the use of miRNA masks which are complementary to the binding sites in the 3’UTR of the target mRNA [15]. This method allows a more specific inhibition of the mRNA targeted by a specific miRNA.

### microRNA replacement therapy

miRNA replacement therapy aims at substitution of tumor suppressive miRNAs expressed at lower levels by using oligonucleotide mimics containing the same sequence as the mature endogenous miRNA. As double stranded miRNA mimics have a much higher potency as single stranded miRNA mimics they are most often used [102]. The guide strand contains a sequence identical to the mature miRNA and the passenger strand sequence is complementary to the mature miRNA. Additionally to miRNA mimics containing the same sequence as the endogenous miRNA, synthetic miRNA precursor mimics with longer sequences are used [103].

## MicroRNA therapeutics

Using a luciferase reporter assay to screen small molecule libraries for a compound that could inhibit the expression of specific oncogenic miRNAs has recently been successful.

### OncomiRs

The expression of microRNA miR-122 is confined to the liver, where it constitutes 70% of the total miRNA population [3]. Within the liver, miR-122 has been implicated in cholesterol and lipid metabolism, and was identified as a regulator for systematic iron homeostasis [7, 8]. Moreover, miR-122 has also been demonstrated to be necessary for the replication and infectious production of hepatitis C virus (HCV). Binding of miR-122 to the 5′ noncoding region of the HCV genome upregulates expression, causing accumulation of viral RNA in liver cells [9]. HCV infection is one of the major causes of liver disease worldwide, including cirrhosis and hepatocellular carcinoma [9]. The essential interaction between miR-122 and HCV suggests that miR-122 could be an excellent therapeutic target for the treatment of HCV infections. Anti-miR-122 is the only miRNA-based treatment tested in human beings so far. In 2010, data from a drug trial of an intravenously delivered anti-miR-122 LNA in chimpanzees were reported [104]. Anti-miR-122 LNA given to chronically infected chimpanzees once a week for 12 weeks led to a reduction in viral load in the serum and the liver. Based on these results, a phase 1 trial in 77 healthy volunteers demonstrated the safety of anti-miR-122 application in humans. In the subsequent phase 2 trial the safety and efficacy of the treatment was confirmed [105]. The discovery of small molecule inhibitors of miR-122 function demonstrates a novel approach to inhibit HCV replication in liver cells [10]. A very recent publication describes the development of an assay for the discovery of small molecule regulators of miR-122, and ultimately HCV therapeutics [106].

miR-21 is an oncogene and therefore frequently highly expressed in solid tumors and hematological malignancies [21, 107–116]. Inhibition of miR-21 resulted in reduced cell proliferation accompanied by increased apoptosis in breast and glioblastoma cell lines [117, 118]. Again by performing luciferase reporter assay an inhibitor of miR-21 has been identified. This agent was able to inhibit miR-21 expression and elicit antitumoral effects [119]. miR-21 transfection leads to the downregulation of PTEN and increased signaling through the PI3K-AKT pathway [34].

Members of the miRNA-29 family (miR-29a, miR-29b, and miR-29c) are known to be highly expressed in normal tissues and downregulated in different types of cancer, including neuroblastoma, sarcoma, glioma, high-risk chronic lymphatic leukemia (CLL), invasive breast cancer, cholangiocarcinoma and lung cancer.(35–40) miR-29a has been shown to reduce invasiveness and proliferation of human carcinoma cell lines.(41) The miR-29 family members also target DNA methyltransferases (DNMT3A and DNMT3B), and can thereby restore patterns of DNA methylation and expression of silenced tumor suppressor genes.(31) We recently showed that inhibition of endogenous miR-29b by stable transduction of a lentiviral vector containing an antisense nucleotide in human lung cancer cells caused increase of inhibitor of differentiation 1 (ID1) and Matrix-Metalloproteinase-9 (MMP9), and enhanced matrigel invasion [120]. On the contrary side, stable overexpression of miR-29b caused decrease of ID1 and MMP9 and significantly decreased invasion [120]. In a further study we observed a reciprocal association between miR-381 and ID1 in lung cancer cell lines and primary adenocarcinomas [121]. Our results also provide first evidence that ectopic expression of miR-381 reduced ID1 mRNA and protein levels, and significantly decreased lung cancer cell migration and invasion.(reviewed in [122]).

The use of antagomiRs against miR-10b in an animal model of breast tumor-bearing mice was associated with reduced metastasis, both *in vitro* and *in vivo*[123]. The silencing of miR-10b with antagomiRs significantly decrease dmiR-10b levels and increased the levels of a functionally important miR-10b target, Hoxd10. The use of this antagomiRs in mice bearing highly metastatic breast cancer cells did not reduce primary mammary tumor growth but markedly suppressed the formation of lung metastases. The therapy was well tolerated by mice.

miR-155 was found to be overexpressed in different types of solid cancers as well as lymphomas [20, 124–133] miR-155 is a negative prognostic factor in pancreatic and lung cancer patients [20, 131]. In malignant glioma the downregulation of the GABA-A receptor was shown to correlate with the grade of the tumor. The knockdown of miR-155 involves the re-expression of GABRA 1 protein *in vivo* and therefore controlling proliferation and signaling pathways regulated by the GABA-A receptor [134].

The inhibition of the MYC-driven miR-9 using a miRNA sponge could reduce the development of lung metastases in a breast cancer mouse model [135]. On the other hand, the inhibition of the tumor suppressive miR-31 with sponge miRNAs in a breast cancer model induced the development of lung metastases [136].

### Tumor suppressor miRNAs

The let-7 family is one of the best described tumor suppressor miRNAs [24, 137–141] and is frequently downregulated in tumor tissue [142]. In xenograft models, tumor burden was reduced by intratumoral delivery of let-7b [143]. By intranasal delivery of let-7a using lentivirus in a lung cancer xenograft model the tumor burden was significantly reduced [57]. One emerging concept for miRNA regulation is based on functional polymorphisms in the target mRNA 3’UTR interfering with miRNA binding and function. Target polymorphisms in the 3’UTR of KRAS interfere with the function of let-7 and are associated with outcomes in breast and lung cancer [144, 145].

The miR-34 family has been reported as direct p53 transcriptional target. Overexpression of miR-34 family member induces apoptosis and cell cycle arrest [146, 147]. The correlation between downregulation of miR-34 and various tumor types has been demonstrated [148–151] miR-34 incorporated in a lipid-based particle was able to block tumor growth in a mouse model of non-small cell lung cancer [57] miR-34a accumulated in the tumor tissue, resulting in downregulation of its direct targets. Similar results were obtained in a second study of non-small cell lung cancer with the delivery of miR-34a or let-7 mimics [57]. Based on these results, miR-34 as a liposomal miR-34 mimic (MRX34, Mirna Therapeutics Inc.) is investigated in clinical trials [152].

miR-16 conjugated to atelocollagen has been shown to reduce bone metastases in a xenografts model of prostate cancer [153]. Atelocollagen is a collagen solubilized by protease with similar physical properties to those of natural, insolubilized collagen [92].

## Conclusions

microRNAs represent critical regulators of tumor cell differentiation, proliferation, cell cycle progression, invasion and metastasis. Based on microRNA arrays various miRNAs have been described as oncogenes or tumor suppressors and many of them are used for diagnosis and as prognostic or predictive tools [122].

Emerging evidence suggests that inhibition of overexpressed oncogenic miRNAs or substitution of tumor suppressive miRNAs could become a novel treatment strategy in cancer therapy. The optimization of the stability of miRNAs, the improvement in delivery systems and targeted drug delivery as well as the understanding and control of off-target effects of miRNA therapeutics are challenges for the future development.
